# Inclusive fitness for in-laws

**DOI:** 10.1098/rsbl.2018.0515

**Published:** 2018-10-10

**Authors:** M. Dyble, A. Gardner, L. Vinicius, A. B. Migliano

**Affiliations:** 1 Jesus College, University of Cambridge, Jesus Lane, Cambridge CB5 8BL, UK; 2 Deparment of Zoology, University of Cambridge, Downing Street, Cambridge CB2 3EJ, UK; 3 School of Biology, University of St Andrews, St Andrews KY16 9TH, UK; 4 Department of Anthropology, University College London, 14 Taviton Street, London WC1H 0BW, UK; 5 Department of Anthropology, University of Zurich, Winterthurerstrasse, Zürich, Switzerland

**Keywords:** kin selection, affines, kinship, inclusive fitness

## Abstract

Cooperation among kin is common across the natural world and can be explained in terms of inclusive fitness theory, which holds that individuals can derive indirect fitness benefits from aiding genetically related individuals. However, human kinship includes not only genetic kin but also kin by marriage: our affines (in-laws) and spouses. Can cooperation between these genetically unrelated kin be reconciled with inclusive fitness theory? Here, we argue that although affinal kin and spouses do not necessarily share genetic ancestry, they may have shared genetic interests in future reproduction and, as such, can derive indirect fitness benefits though cooperating. We use standard inclusive fitness theory to derive a coefficient of shared reproductive interest (*s*) that predicts altruistic investment both in genetic kin and in spouses and affines. Specifically, a behaviour that reduces the fitness of the actor by *c* and increases the fitness of the recipient by *b* will be favoured by natural selection when *sb* > *c*. We suggest that the coefficient of shared reproductive interest may provide a valuable tool for understanding not only the evolution of human kinship but also cooperation and conflict across the natural world more generally.

## 1. Background

Hamilton's rule provides a framework for understanding the evolution of altruism among genetically related individuals [1–3]. It states that an altruistic act will be fitness enhancing if the cost to the altruist (*c*) is less than the benefit to the recipient (*b*) multiplied by the relatedness of the altruist to the recipient (*r*). This leads to the prediction that organisms will benefit from recognizing and preferentially cooperating with kin [2,3]. In humans, this appears to be the case, with kinship forming a cornerstone of social, economic and political life across human societies.

However, human kinship comprises not only genetic kin but also kin through marriage—our affines (in-laws) and spouses. Although the importance of cooperation among affinal kin has frequently been noted by anthropologists [4–6] cooperation among affines has typically been either regarded as inconsistent with kin selection [7], or described without reference to evolutionary theory. Hughes [8], however, argued that even if affinal kin are not genetically related, they may have a shared genetic interest in the next generation, a hypothesis empirically tested by Burton-Chellew & Dunbar [9]. Here, we extend these previous studies, developing a general framework for estimating the degree of shared reproductive interest that individuals have in their social partners.

The central logic of inclusive fitness theory is that when an altruist's genetic kin reproduce, they propagate genes that are identical by descent to those carried by the altruist [2,10]. However, in sexually reproducing species, reproduction propagates both an individual's genes and the genes of its reproductive partner. As such, the indirect fitness returns that an altruist ultimately gains from an altruistic act will be determined by its genetic relatedness both to the recipient and to the recipient's reproductive partner. For example, while in the absence of inbreeding an individual may not be genetically related to her brother's wife, she will, assuming monogamous mating, be related to her brother's wife's offspring by *r* = 0.25. The altruist therefore has a genetic interest in her brother's wife's reproduction. Here, we propose a coefficient that captures the shared reproductive interest between individuals.

## 2. Estimating shared reproductive interests

In order to measure the fitness benefits that individuals derive from social interactions with kin of all kinds (genetic, affinal and spousal), we propose a coefficient of ‘shared reproductive interest’ (*s*). This coefficient measures the genetic interest an altruist has in the future offspring of another individual, expressed relative to its genetic interest in its own future offspring. A derivation of *s*, starting from Hamilton's rule [11], is set out below.

Let a focal individual A (the actor) interact with a social partner B (the recipient) such that the number of offspring the actor successfully rears to adulthood is reduced by *c* and the number of offspring the recipient successfully rears to adulthood is increased by *b*. Assuming no other consequences, the condition for this act of altruism to be favoured is that it increases the actor's inclusive fitness, i.e.2.1
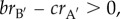
where *r*_A′_ is the relatedness of an actor to its own offspring and *r*_B′_ is the relatedness of the actor to the offspring of the recipient. Note that the relatedness of the actor to its own offspring is simply the average of the relatedness of the actor to itself and the relatedness of the actor to the actor's mate, with whom those offspring will be produced. That is:2.2
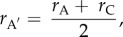
where *r*_A_ is the relatedness of the actor to itself (which, by definition, is 1) and *r*_C_ is the relatedness of the actor to the actor's mate. Likewise, the relatedness of the actor to the recipient's offspring is given by the average of the relatedness of the actor to the recipient and the relatedness of the actor to the recipient's mate, with whom those offspring will be produced. This yields2.3
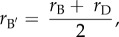
where *r*_B_ is the relatedness of the actor to the recipient and *r*_D_ is the relatedness of the actor to the recipient's mate. The measures of genetic relatedness (*r*) that constitute *r*_B′_ and *r*_A′_ can be defined using either pedigree estimates or genetic markers. Both the ‘actor's mate’ and the ‘recipient's mate’ may refer to a specific individual, or alternatively to an appropriately weighted set of all possible mating partners in cases where there is uncertainty as to this individual's identity (see the electronic supplementary material, methods). Given the above, the condition for the act of altruism to be favoured is2.4
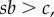
where2.5
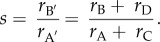
Here, *s* is the actor's inclusive fitness valuation of the recipient's offspring expressed relative to that of the actor's own offspring and is defined as conditional upon an act of altruism taking place. While *r* is a measure of genetic equivalence, *s* is a measure of reproductive equivalence.

**Figure 1.**
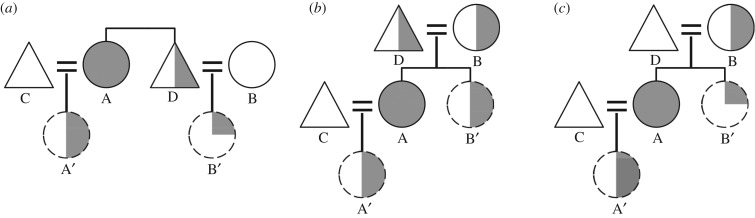
Shared reproductive interest of a female altruist A in a recipient B who is either her sister-in-law (*a*) or mother (*b*,*c*). Horizontal ties represent siblingship, vertical ties represent parenthood and equals signs represent reproductive partnerships. Circles are female, triangles are male. The proportion of a symbol that is shaded represents the coefficient of relatedness (*r*) to A. A′ and B’ (dashed circles) are the hypothetical future offspring of A and B. The relatedness of A to B is *r* = 0.5 in panels (*b*) and (*c*) and *r* = 0 in panel (*a*). The shared reproductive interest of A in B is *s* = 0.5 in panels (*a*) and (*c*) and *s* = 1 in (*b*).

To illustrate the application of the coefficient of shared reproductive interest *s*, consider the relative inclusive fitness interest that women have in their brothers' wives. Although *s* can incorporate both paternity uncertainty and inbreeding (electronic supplementary material, table S1), in this example we assume a non-inbred population characterized by monogamous mating. In such a setting, even though a woman (individual A) is genetically unrelated to her brother's wife (individual B), the former will nevertheless be related to the latter's offspring by *r* = 0.25 (i.e. B's offspring will be A's nieces and nephews). As such, the reproduction of individual B is half as effective at propagating A's genes as is A's own reproduction. Accordingly, *s*_AB_ = 0.5 (figure 1*a*). It is worth noting that the shared reproductive interest (*s*) between two individuals is not necessarily symmetrical. For example, while A is genetically related to the offspring of her brother's wife, the reverse is not true—A's brother's wife will be genetically unrelated to the offspring of A (e.g. while *s*_AB_ = 0.5, *s*_BA_ = 0).

Next, consider A's relatedness to her mother. While A's genetic relatedness to her mother is *r* = 0.5, her reproductive interest in her mother depends upon who her mother reproduces with. In a strictly monogamously mating population, A's mother will mate with A's father, producing full siblings that are as closely related to A as her own offspring. In this case, the shared interest of A in her mother will be *s* = 1 (figure 1*b*). In a non-monogamous population in which A's mother mates with an individual genetically unrelated to A, the resulting offspring will be A's half-siblings (*r* = 0.25) and A will be *s* = 0.5 to her mother (figure 1*c*). Typical *r* and *s* values for some standard classes of kin are provided in table 1.

**Table 1.** Relatedness of an altruist to various consanguineal and affinal kin according to *r* and *s*. The altruist is assumed to be genetically unrelated to its own mate, with both individuals from a non-inbred population. See electronic supplementary material, table S2 for an extended version.

recipient	*r* to recipient	*r* to recipient's mate	*s* to recipient
*consanguines (genetic kin)*			
full sibling	0.5	0	0.5
parent (mating with other parent)	0.5	0.5	1
parent (mating with non-parent)^a^	0.5	0	0.5
niece/nephew	0.25	0	0.25
cousin	0.125	0	0.125
*affines*			
spouse	0	1	1
sibling's spouse	0	0.5	0.5
spouse's sibling	0	0	0

^a^Assumes that this non-parent is *r* = 0 to the altruist.

## 3. Discussion

The coefficient of shared reproductive interest defined here provides a framework for estimating the fitness benefits that individuals can derive from social interactions with spouses and affinal kin (in-laws) as well as genetic kin. Although we refer to in-laws, a human-specific term, our analysis can be applied to all notions of extended kin through mating. We argue that shared reproductive interest represents a concept that not only is theoretically consistent with inclusive fitness theory and its subsequent derivations and expansions [12,13] but also lends itself to the empirical study of social relations among genealogical and affinal kin, extending the work of Hughes [8,9].

Unlike genetic relatedness, shared reproductive interest is not usually symmetrical. For example, while a mother has an interest in the reproduction of her son's wife, the reverse is not true, an asymmetry that has been advanced for the evolution of the menopause [14]. Similarly, the increased shared interest that an actor has in its mother's offspring if she reproduces again with the actor's father (as outlined above) is the basis of the ‘monogamy hypothesis’ for the evolution of eusociality [15,16]. Our coefficient also allows the incorporation of uncertainty about future reproduction, variation in which has been argued to underlie differences in human marriage and inheritance practices including monogamy [17] and avuncular inheritance [18,19]. Although we have not considered different classes of individuals (e.g. young versus old, reproductive versus non-reproductive), this can be incorporated by considering reproductive value [20], and the framework could be extended from the dyadic one considered here to consider the relatedness of the altruist to multiple social partners.

The idea that kinship can explain and predict instances of altruistic behaviour is one of the most important insights in evolutionary theory and can be traced back to Darwin himself [21]. Although typically applied only to genetical kin, we argue that the framework of inclusive fitness can be extended to understand the evolution of altruism toward reproductive partners common across the natural world and, in the case of humans, to affinal kin and spouses. Hamilton [2, p. 16] famously wrote that, on the basis of inclusive fitness theory, ‘we expect to find that no one is prepared to sacrifice his life for any single person but that everyone will sacrifice it when he can thereby save more than two brothers, or four half-brothers or eight first cousins'. According to our definition of the coefficient of shared reproductive interest*,* we might also add ‘or two daughters-in-law or eight cousin's spouses’.

## Supplementary Material

Supplementary Methods

## Data accessibility

This paper has no associated data.
