# Supplementary material for: Inclusive fitness for in-laws
Source: Biol Lett. 2018 Oct 10;14(10):20180515. doi: 10.1098/rsbl.2018.0515 (PMC6227869; doi:10.1098/rsbl.2018.0515)
Supplement: Supplementary Methods [file rsbl20180515supp1.pdf]

Supplementary Methods: Inclusive fitness for in-laws

M. Dyble, A. Gardner, L. Vinicius, A.B. Migliano

Incorporating uncertainty in mating

As stated in the main text, relatedness to the "actor's mate" (r_C in equations 2.2 and 2.5) may refer to a specific individual, or alternatively to an appropriately-weighted set of all those possible mating partners with whom the actor would have produced the *c* offspring that they have lost on account of their altruism. Where it represents such a weighted set of possible mating partners, r_C can be defined as:

$$r_C = \Sigma\ i \in I_A\, p_{A,i}\, r_{A,i}$$

where I_A is the set of the actor's possible mates, with $p_{A,i}$ being the probability that individual $i \in I_A$ would have been the parent of a particular offspring lost by the actor, and $r_{A,i}$ being the actor's relatedness to this possible mating partner).

Similarly, the relatedness of the actor to the recipient's mate (r_D in equations 2.3 and 2.5) can be defined either as a specific individual or to a weighted set of all possible mating partners such that:

$$r_D = \Sigma\ i \in I_B\, p_{B,i}\, r_{B,i}$$

where I_B is the set of the recipient's possible mates, with $p_{B,i}$ is the probability that individual $i \in I_B$ would have been the parent of a particular offspring lost by the recipient, and $r_{B,i}$ being the actor's relatedness to this possible mating partner of the recipient. Defining relatedness to the actor and recipient's mates as above allows for the incorporation of extra-pair mating, the dissolution of pair-bonds, and, in the case of human kinship, divorce.

Inbreeding and s

The formulation of *s* given in Equation 2.5 in the main text can incorporate inbreeding. Consider the examples given in Table S1. In the absence of inbreeding, an individual will have a genetic relatedness of $r = 0.5$ to his sister, and a shared reproductive interest of $s = 0.5$. Should his sister mate with his cousin of $r = 0.125$ (as in 'Cousin marriage 1' in Table S1), then his interest in his sister's offspring will increase, relative to his interest in his own offspring, leading to an estimated shared reproductive interest of $s = 0.625$. Conversely, if his sister mates with an unrelated individual and he mates with his cousin, then his relative interest in his sister's offspring in lower ($s = 0.444$). If both he

and his sister mate with their cousins, then the degree of shared reproductive interest is $s = 0.556$.

Table S1: The shared reproductive interest of an actor in a full sibling given various scenarios of inbreeding or cousin marriage.

Example	r_A	r_B	r_C	r_D	$r_{A'}$	$r_{B'}$	s
No inbreeding (full sibling)	1	0.5	0	0	0.5	0.25	0.5
Cousin marriage 1	1	0.5	0	0.125	0.5	0.3125	0.625
Cousin marriage 2	1	0.5	0.125	0	0.5625	0.25	0.444
Cousin marriage 3	1	0.5	0.125	0.125	0.5625	0.3125	0.556
Past inbreeding	1	0.6	0	0	0.5	0.3	0.6

Table S2: Extended calculation of coefficients of shared reproductive interest in Table 1.

Example	r_A	r_B	r_C	r_D	$r_{A'}$	$r_{B'}$	s
Full sibling	1	0.5	0	0	0.5	0.25	0.5
Parent (mating with other parent)	1	0.5	0	0.5	0.5	0.5	1
Parent (mating with non-parent)	1	0.5	0	0	0.5	0.25	0.5
Niece/nephew	1	0.25	0	0	0.5	0.125	0.25
Cousin	1	0.125	0	0	0.5	0.25	0.125
Spouse	1	0	0	1	0.5	0.5	1
Sibling's spouse	1	0	0	0.5	0.5	0.25	0.5
Spouse's sibling	1	0	0	0	0.5	0	0
